# “It's my frenemy”: A qualitative exploration of knowledge and perceptions of fentanyl use during the COVID-19 pandemic in people who use drugs at a syringe services program in Philadelphia, PA

**DOI:** 10.3389/fpubh.2022.882421

**Published:** 2022-07-22

**Authors:** Sarah Bauerle Bass, Patrick J. A. Kelly, Sphoorti Pandit-Kerr, Jenine Pilla, Katherine Morris, Erin Larsen, Jennifer P. Wisdom, Phillip R. Torralva

**Affiliations:** ^1^Department of Social and Behavioral Sciences, Temple University College of Public Health, Philadelphia, PA, United States; ^2^Risk Communication Laboratory, Temple University College of Public Health, Philadelphia, PA, United States; ^3^CODA, Inc., Portland, OR, United States

**Keywords:** fentanyl, overdose, COVID-19, qualitative in-depth interviews, harm reduction

## Abstract

**Background:**

Exacerbated by the COVID-19 pandemic and the proliferation of fentanyl and fentanyl analogs, overdose deaths have surged in the United States, making it important to understand how individuals who use drugs experience and perceive the risks of fentanyl use and how it has changed during the COVID-19 pandemic.

**Methods:**

Twenty clients from a Philadelphia syringe services program completed a questionnaire and in-depth interview about their fentanyl experiences from January to March 2021. These interviews were transcribed and analyzed using thematic analysis methods.

**Results:**

Sixty percent of participants were female and racial/ethnic minority. Participants indicated they believed fentanyl use accounted for most Philadelphia opioid-related overdoses and understood that fentanyl was different from other opioids. Fentanyl use was characterized as “all-consuming” by taking over lives and inescapable. While most perceived their risk of fentanyl overdose as high, there was low interest in and reported use of harm reduction strategies such as fentanyl test strips. The COVID-19 pandemic was noted to have negative effects on fentanyl availability, use and overdose risk, as well as mental health effects that increase drug use.

**Conclusions:**

The divide between perceived risk and uptake of protective strategies could be driven by diminished self-efficacy as it relates to acting on and engaging with resources available at the syringe services program and represents a potential intervention target for harm reduction intervention uptake. But the COVID-19 pandemic has exacerbated risks due to fentanyl use, making an effective, accessible, and well-timed intervention important to address the disconnect between perceived overdose risk and use of preventive behaviors.

## Introduction

Opioid overdose is the most common cause of accidental death in American adults ages 18-50, and deaths involving synthetic opioids were nearly 12 times higher in 2019 than 2013 (1). The United States' fentanyl-driven opioid crisis has claimed the lives of nearly 250,000 people between 2011 and 2019 (2–4) and an additional 75,673 opioid related deaths occurred in the year prior to April 2021–an ~35% increase from the prior year (5) This surge in fatal overdoses across the US is fueled by the rising prevalence of fentanyl and other adulterants which have largely replaced heroin alone in many drug US markets. In Philadelphia, the location of this study, fentanyl and fentanyl analogs are increasingly used either alone or in combination with other drugs (6). In 2020, 94% of opioid deaths involved fentanyl or fentanyl analogs, making fatal and non-fatal opioid overdose a significant public health crisis in Philadelphia (7). The increase in opioid overdose has only been exacerbated by the COVID-19 pandemic (8), in part because COVID-19 mitigation strategies, such as lockdowns and social distancing, may decrease the likelihood of overdose reversal with naloxone (9).

The prevalence of fentanyl use, especially when used in combination with other drugs such as stimulants (e.g., methamphetamine, cocaine), has also increased overdose rates, contributing to significant mortality increases in all types of drug use (10, 11). While some may intentionally use fentanyl and other substances, some may not realize they are using fentanyl. A 2019 analysis of approximately 1 million patients' urine drug test (UDT) specimens across the U.S. showed that the fentanyl-positive test rate increased by 1,850% among cocaine positive and 798% among methamphetamine-positive UDT results between January 2013 and September 2018, where the majority of individuals tested were unaware of their fentanyl use (12, 13). Fentanyl and fentanyl analogs, like all opioids, cause respiratory depression, and can have rapid lethal effects on respiration from vocal cord closure and severe rigidity of the chest wall muscles (14–16). Although naloxone reverses respiratory depression caused by opioid overdose, clinical and animal studies (17, 18) on overdose from fentanyl are suggested to require two to three times the current recommended dose for an opioid-related overdose (19). This has particular significance in the context that fentanyl produces a quick and potentially more dangerous high that also requires more frequent use (20), and multiple surveys in various opioid endemic regions in the U.S demonstrate over half of the individuals who use illicit opioids do not have naloxone readily or routinely available (21–23).

A number of community-based studies demonstrate there is awareness among people who use opioids that fentanyl use increases overdose risk (22, 24), and that people who use opioids engage in strategies to reduce the effects of health-harming substances including overdose (25). Exchanging syringes, using drugs with a partner, carrying naloxone, using fentanyl test strips, and taking tester shots of small amounts of a drug before large doses are well documented health-protective mitigation strategies (25–27). These harm reduction measures have developed and adapted to meet the immediacy of emergent health threats, as was observed with response efforts to the HIV/AIDS epidemic (28). Indeed, the COVID-19 pandemic and the increasing prevalence of fentanyl and fentanyl adulterants in overdose deaths changed the landscape in which substances are consumed, which necessitated the evolution of health-protective behaviors to reduce harm. The urgency of understanding how harm reduction strategies adapt is underscored by the fact that fentanyl overdose causes the rapid onset of airway obstruction and unconsciousness, a high risk for mortality and morbidity without immediate intervention from first responders (14, 16, 27).

Despite this significant public health crisis (1–7), the public health community is only beginning to understand how people who use drugs perceive of the risks of fentanyl and, more importantly, how they would like to receive critical information in a medium that will increase harm reduction practices, and how the COVID-19 pandemic has affected these perceptions. To address this gap, this study used in-depth interviews with clients of a large urban syringe services program (SSP) that provides psychosocial and medical services to people who use drugs located in the Kensington neighborhood of Philadelphia–the epicenter of opioid use and unintentional overdose in Philadelphia (7). Harm reduction practices are developed and disseminated throughout drug-using communities *via* SSPs (26), making them important to understanding experiences with fentanyl and harm reduction practices. The following a priori research questions were established:

1. What were participants' personal knowledge of and experience with fentanyl?
2. What were participants' concerns about fentanyl and what additional information did they want to know?
3. How did participants identify fentanyl in substances they use, and what was their experience using fentanyl test strips?
4. How did the COVID-19 pandemic affect substance use, fentanyl use, and overdose?

## Methods

### Participants and setting

In-depth interviews were conducted with clients of Prevention Point Philadelphia in Kensington. Prevention Point Philadelphia distributed nearly 6 million syringes between 2019 and 2020–making this organization the largest syringe services program in the United States and an appropriate study site to address the research questions, according to the organization's Executive Director (2021). Clients (*n* = 22) were approached by study staff (PJAK, JP) while they were onsite to access services, such as syringes, case work, mail or medical services. This occurred on different days and times to correspond to when services were provided to ensure a cross-section of different types of clients. Clients were asked if they were interested in participating and 21 provided informed consent. One participant was interviewed twice, but only their first interview was retained for analysis. When available, SSP staff facilitated introductions between clients and study staff. Eligible participants included clients 18 years of age or older who self-reported current drug use.

### Data collection

Participants completed a brief demographic form prior to the interview. Demographics included age, gender, race, education level, and financial and housing stability. Four items informed by the expertise of the authors (29–31) asked participants to state agreement from 0 “strongly disagree” to 4 “strongly agree” to assess fentanyl beliefs. Items were: 1. Fentanyl causes most of the opioid overdoses in Philadelphia; 2. A small amount of fentanyl can lead to hospitalization, overdose, or death; 3. Fentanyl can be found in substances people use other than heroin; and 4. More than one dose of naloxone/Narcan can reverse a fentanyl overdose.

Interviews were conducted as part of a National Institute on Drug Abuse funded study aimed to understand the effect of COVID-19 on people who use drugs. Participants were asked to discuss experiences with fentanyl during the interview. Questions assessed whether and how participants sought out or avoided fentanyl, and interviewers probed to assess use of fentanyl test strips. Interviewers also assessed perceptions of whether and how the increasing prevalence of fentanyl changed the types of drugs participants used since the emergence of COVID-19, and whether participants knew enough about fentanyl and concerns about fentanyl use broadly.

Interviews were conducted by trained study personnel (PJK, JP, male and female, respectively) who have significant experience working with people who use drugs, audio recorded, and transcribed verbatim. Data were collected January through March 2021. Participants provided informed consent and received a $25 gift card at interview completion. The Temple University Institutional Review Board approved this research (27637), and Prevention Point Philadelphia approved the study taking place at their location.

### Analyses

Summary thematic analysis determined that thematic saturation had been reached after 20 interviews, so no further interviews were completed. Descriptive statistics for categorical (i.e., frequencies and proportions) and continuous (i.e., mean and standard deviations) data are presented for sociodemographic variables (Table 1). Inferential statistics were not conducted given the sample size. SPSS statistics for Macintosh version 25 was used. Transcripts were bound to the interview portion that assessed fentanyl experiences, as this was relevant to answering the research questions. Guided by the principles of Applied Thematic Analysis, (32) two trained analysts reviewed each transcript section, generated patterns, and met to reach thematic consensus. Applied Thematic Analysis is a qualitative evaluation method not tied to a theory and is appropriate for formative work that aims to describe themes found in data. In this study, Braun and Clarke's (33) suggested stages were used, including becoming familiar with the data, generating initial codes, searching for themes, and deriving meaning. A codebook was developed and the analysts used this codebook to identify quotes to derive meanings across interviews. Any discrepancies were decided through discussion. Results are presented by research question (sample quotes are presented in Table 2).

**Table 1:** Demographics.

	**N (%)**
Gender
Male	8 (40%)
Female	12 (60%)
Race/Ethnicity
Black/African American	7 (35%)
White	8 (40%)
Latino/a	3 (15%)
Multiracial	1 (5%)
Declined	1 (5%)
Highest level of education
Less than High School	7 (35%)
High School or GED	9 (45%)
Technical, vocational, community college	1 (5%)
Some College	1 (5%)
College degree or above	2 (10%)
Monthly income*
$0–$500	10 (50%)
$501–$1,000	8 (40%)
$4,000+	1 (5%)
Exchanged sex for money or other things
No	13 (65%)
Yes	7 (35%)
Living situation in last 6 months
House/Apt own or rent	3 (15%)
Partner who pays rent/mortgage	1 (5%)
Friends/Family who pay rent/mortgage	4 (20%)
Shelter	3 (15%)
Street, Park, Abandoned Building, Car etc.	9 (45%)
	Mean (SD)
Age (range: 23–62)	43.3 (10.7)

^*^Total is <20 due to missing data.

**Table 2:** Selected quotes by research question.

**RQ#1: What are participants' personal knowledge and fentanyl use experiences**	“I'm completely addicted to it. And it's really hard to get off of it. And the fear of the sickness and everything else is really a deterrent for getting clean for everybody.” (Participant #2)
	“It's my frenemy. It picked me up when I'm down, but it put me down more. It's the only person that was ever there for me. When I cry it pats me on my back, but when I'm down they're pushing me down more. It took everything from me.” (Participant #6)
	“So, I like got a relationship with it. And then I tried to leave it. And I can't, and now I'm just out here by myself and I can't stop. < Crying> It's just so stressful because I don't want this lifestyle. Like, I'm a whole nerd, like I'm really a nerd. I like just learning, going to school and stuff. But something happened. Just it took me, like it grabbed me and it held me. It got me in a headlock. Like, I be crying because I don't want to do it, but I can't help it. Like right now I'm going through withdrawal on purpose because I'm really trying to like stop. But like, I just can't.” (Participant #6)
	“It's like yo, it's the devil like those blue bags are like it's the devil himself on earth. I swear. I swear. People will kill their own love for that little blue bag. Like the stuff I see, that happened to me.” (Participant #6)
	“It is the worst thing that I ever touched. Umm it's horrible. The withdrawal from it is horrible. You need it every few hours. Like, it's just, it's the devil, like, I want to get off it so bad and I just there's nothing out here right now that helps with the withdraw.” (Participant #10)
	“I've been using for 8 years now. I'm 31. I'm going from regular heroin to the fentanyl and it's, it's been um, it's terrible. I've seen a lot of people die. I've overdosed a lot of times. It's a lot stronger, scary. And it's a day to day struggle, like it's up the hill, life threatening struggle, you know? Um, it's scary. It's scary.” (Participant #19)
	“There's so much…It's traumatic. It's very dramatic. Like just yesterday, I had two guys pull a razor out over to take the money back that I made. Because I needed, I was, put myself in a situation like that because I need the fentanyl. Because it makes me sick without it. So yeah, it makes me sick emotionally too. I'm tired of this.” (Participant #19)
**RQ2: What are participant concerns about fentanyl and what additional information do they** **want to know?**	“I didn't originally use fentanyl and no one explained to me that when I went from regular heroin to fentanyl how bad and hard fentanyl is to get off. Yeah, and how like methadone does not touch it for a period of time.” (Participant #3)
	“I just wish I wasn't doing it.” (Participant #1)
	“And you have to be on such a high dose, just to show regular. It's just it's gonna be the fight of your life. And after 3 days, that's it. You're addicted doing it 3 days in a row. I was unaware. And before I knew I was addicted, and now it's my life revolves around it.” (Participant #4)
	“Yeah (knows about fentanyl) through experience but not like through a doctor telling me and giving me showing me that this is what it really is, this is what it's gonna take to get off. This is what it does to your body. You never know your hands swell up, your feet swell up. You know, you can't sleep, restless syndrome, you're throwing up, your stomach hurts. You can't eat. They don't. None of that is being said, all you want to do is addict to it. So, we need way more understanding and information on it.” (Participant #4)
	“I wish I knew that it would fucking do this to me. Like it's different when you smoke pot for the first time, you know what I mean?” (Participant #6)
	“Um that was my first time I've ever, ever overdosing off anything. Um, I've been using drugs for years, you know, and I know this drug is different. I ain't never seen no shit that killed you like this. It's mind numbing, certainly.” (Participant #8)
	“I know it's dangerous to us. I know all that stuff. But at this point, it's like my body needs it. So, every day it's like, every time I do it, it's like, alright, I hope I make it out of this one. I hope I make it out of this one.” (Participant #10)
	“Sometimes I be thinking what if fentanyl's not good enough anymore. What they gonna come out with next. Cuz it was regular heroin, from heroin it went to fentanyl, you know, who knows what's going to come out next. I don't want to do it. God forbid. What about it'll probably be the worse of the worse you know. it'll probably be so strong that it could end up more people then when people dying now.” (Participant #16)
**RQ#3: How do participants identify fentanyl in substances they use, and what is their experience using fentanyl test strips?**	“They're (test strips) good to have. But at this point, I need fentanyl.” (Participant #10)
	“You know what's crazy, I just talked to the doctor about that. And he was telling me I how to get some. Yeah, he told me you can test the drug and see. Yeah, cuz I heard about them, I never used them myself.” (Participant #14)
	“Sure (use test strips). Because I'd rather be more safer. Or even though I know that's not a good thing to say or do by you know, there's other stuff people putting in the drugs nowadays that I don't want to be hurt, either.” (Participant #16)
**RQ#4: How has the COVID-19 pandemic affected substance use, fentanyl use, and overdose?**	“More fentanyl overdoses since the pandemic, people aren't working, people are depressed people are constantly overwhelmed and flooded with all this COVID-19 information. Now you can't go to work. Now you have to stay inside your house. Now you're stuck with your thoughts? Well, you know, it's a scary place to be, especially for people that were already addicts being clean, and then they relapse. And that's why I think there's more overdoses during the pandemic.” (Participant #2)
	“I mean, when COVID hit they changed the drugs—the suppliers. It seems like when COVID hit it seem like its more tranquilizer than it is fentanyl…I think it might be cheaper.” (Participant #3)
	“Yeah, I think it has…cuz people are stressed out and they want their relief because they don't want to deal with everything that's coming on, like having to worry about feeding your family and paying your bills. People want some type of an escape, a relief. So, I think it has been made available. To drug dealers they want to capitalize on it, cuz it's becoming more in demand because they realized people need escape, even if it's for a couple hours. So, I think the availability has been made, you know, to suit because of demand. Because of the stress that's going on.” (Participant #9)

## Results

Sixty percent of participants were female and identified as racial/ethnic minority. The mean age was 43.3 (SD 10.7). Most (90%) were homeless and earned < $1,000 monthly. Participants were aware of differences between fentanyl and other opiates, and that substances like cocaine could also be adulterated with fentanyl (mean = 3.65, SD.49). Fentanyl use was believed to cause hospitalization, overdose, and death (mean = 3.55, SD.51) and account for most Philadelphia opioid-related overdoses (mean = 3.40, SD.68). Slightly fewer reported thinking that more than one dose of naloxone would need to be used to reverse an overdose due to fentanyl (mean = 3.2, SD.83).

### Research question 1: What is your personal knowledge and fentanyl experience/use?

Participants stated they were aware that fentanyl was a different substance from heroin and other opioids. They conveyed that fentanyl was stronger, that they became more easily dependent on it compared to heroin, and that fentanyl was distinguishable from heroin by the “different high” experienced with fentanyl use. Personal experiences with fentanyl were characterized as a struggle between awareness of its dangers and not liking the sensation of tiredness after use but needing to continue due to severe withdrawal symptoms. Participants also shared being unable to stop fentanyl use despite negative experiences of overdose, cravings, and fear of death. One participant described how quickly she became dependent on fentanyl and the negative physical effects of its use:

“*And before I knew I was addicted, and now my life revolves around it...I can't (stay away from it)…This is what it does to your body. You never know your hands swell up, your feet swell up. You know, you can't sleep, restless syndrome, you're throwing up, your stomach hurts. You can't eat*.” (Participant 4)

Another participant said, “*It's like yo, it's the devil. Like those blue bags are like it's the devil himself on earth. I swear. I swear. People will kill their own love for that little blue bag*.” (Participant 6)

Some participants personified their relationship to fentanyl. One participant called fentanyl her “frenemy”. She said, “*It picked me up when I'm down, but it put me down more. It's the only person that was ever there for me. When I cry it pat me on my back, but when I'm down* [it's] *pushing me down more. It took everything from me.”* She went on to say, “*I like got a relationship with it. And then I tried to leave it. And I can't. And now I'm just out here by myself and I can't stop*.” (Participant 6) Most participants expressed the belief that substances such as cocaine and heroin can be adulterated with fentanyl and reported a belief that fentanyl in Philadelphia was increasingly cut with xylazine (called “tranq” in Philadelphia) since the beginning of the COVID-19 pandemic. Additionally, participants stated they knew naloxone was typically used to help with opioid/heroin overdoses, but believed it was not as effective for fentanyl overdoses.

### Research question 2: What were participant concerns about fentanyl and what additional information did they want to know?

For some participants, there appeared to be a disconnect between understanding of the adverse effects and personal concerns about actual use. Statements such as “*It's like, it's dangerous. If you misuse it you can die*,” (Participant 3) and “*It's [fentanyl] bad for you*” (Participant 7) indicated knowledge of the dangers of fentanyl; however, these were often followed by a lack of concern about personal adverse effects from use with statements such as “*Not* (concerns about fentanyl) *for the most part*.” (Participant 7)

Conversely, other participants shared their personal concerns about fentanyl use in statements such as “*I just wish I wasn't doing it*” (Participant 1) and “*I'm concerned what it's doing my body*,” (Participant 5) while expressing a lack of knowledge about consequences of long-term use. Most of these participants said they wished they had known more about fentanyl's effects on the body both in the short and the long term before they started using it. For example, one participant said: “*I didn't originally use fentanyl, and no one explained to me that when I went from a regular heroin to fentanyl how bad and hard fentanyl is to get off* .” (Participant 3) Another participant indicated that despite long term substance use, fentanyl was different. They said:

“*That was my first time I've ever, ever overdosing off anything. I've been using drugs for years, you know, and I know this drug is different. I ain't never seen no shit that killed you like this. It's mind numbing, certainly*.” (Participant 8)

Participants said that access to concrete information that could be seen and read might result in people taking the risks of fentanyl use more seriously. Participants suggested sharing information based on studies about what really happens in the body and consequent symptoms of fentanyl use, why it began to be circulated, how long it takes to wear off, and the severity of withdrawal as ways to increase awareness about fentanyl. Once participant said:

“*I don't even know all the symptoms, like if I can get any more informative information, meaning something that I can read and see. Or speak to doctors and nurses or even, you know, students… anyone that can give me facts, not just the drug aspect of it. The real thing. Yeah. Well, what the studies have shown and what's really happened, you know what I mean, it'd be way more helpful to people because they would take it more seriously. See, the fact of everything is not word of mouth.”* (Participant 4)

### Research question 3: How do participants identify fentanyl, particularly their knowledge and experience using test strips?

Most participants shared knowing that fentanyl test strips could identify the presence of fentanyl in substances but did not use them. For example, one participant said, “*I know you can take the drugs if you use the test strips to see if fentanyl's in it. I don't use test strips. I just don't*.” (Participant 1) Only 20% of the participants interviewed expressed either an interest in and/or experience with using the test strips including knowing where to obtain them and using them as a safety measure: “*I just got ‘em last week and used it (test strips) and it was good*.” (Participant 8). One participant (16) learned about test strips from people at Prevention Point and felt strips could help them “*be more safer*”, yet this participant indicated they did not use them. Another said they had some in their purse but did not use them. Not all participants responded directly to this question and one participant shared that they identified fentanyl by smell based on experience from long term use, making test strips unimportant: “*Once you do it all the time and you've been doing it for a while you know you can smell it. You can smell it. You put the water on there. It's got a certain smell*.” (Participant 3) Another indicated that they did not use them because they take too long. They said: “*No, they'd* (fentanyl test strips) *be a pain in the ass…Because as soon as I get the bag of dope, I want to snort that before the cops come.”* (Participant 17). Similarly, another participant indicated they knew about test strips and acknowledged that having test strips was good but felt that the need to use fentanyl made them irrelevant: “*They're good to have. But at this point, I need fentanyl*.” (Participant 10).

### Research question #4: How has the COVID-19 pandemic affected substance use, fentanyl use, and overdose?

Finally, many participants discussed substance use, fentanyl use, and overdose in the context of what was happening during the COVID-19 pandemic and why overdose risk and substance use changed. Some discussed the economic hardships people were facing, especially those who were trying to get money through hustling, sex work or other means, and how these financial pressures increased substance use. One participant shared a belief that COVID-19 mitigation strategies fostered a dangerous environment for people with existing drug use dependencies because of the mental distress brought about by the crisis. They said:

“*Now you can't go to work. Now you have to stay inside your house. Now you're stuck with your thoughts. Well, you know, it's a scary place to be, especially for people that were already addicts...That's why I think there's more overdoses during the pandemic.”* (Participant 2

Another participant said that dealers exploited people's fears, vulnerability, and desire to use drugs to cope with pandemic stressors by pushing more dangerous drugs on the street or to change the types of drugs that were available for people which increased the risk of overdose. They said:

“*People want some type of an escape, a relief. So…drug dealers they want to capitalize on it, cuz it's becoming more in demand because they realized people need escape, even if it's for a couple hours. So, I think the availability has been made, you know, to suit because of demand. Because of the stress that's going on*.” (Participant 9)

## Discussion

The results of this study provided insight into the lived experiences about the risks associated with fentanyl use in a high overdose-risk population from a large SSP during the COVID-19 pandemic. Our sample characterized fentanyl use as “all-consuming” and nearly inescapable from daily life. But a chasm between high perceived risks of fentanyl and low interest in and reported use of harm reduction strategies, such as fentanyl test strips, was identified. Given that this sample drew from a well-resourced syringe services program, it is noteworthy that participants reported underutilization of overdose prevention strategies despite the collective recognition that fentanyl posed life-threatening risk. The divide between perceived risk and uptake of protective strategies could be driven by myriad interrelated factors. If thought of in a risk environment framework, as proposed by Rhodes, there are individual factors that must be considered but also social and physical factors from the environment in which drug use occurs that increase or decrease the chances of harm during drug use. (34) Given participants' characterization of fentanyl dependency, it could be that the need to use drugs to maintain wellness superseded prioritization of protective strategies like use of fentanyl test strips, despite their availability. Further, diminished self-efficacy as it related to acting on and engaging with resources available at the syringe exchange may have contributed to the underutilization of protective strategies reported by the sample, illustrating the dynamic interaction of individual and environmental factors. This represents a potential intervention target for harm reduction intervention uptake, (35) and draws attention to the immediacy and importance of increasing access to structural harm reduction strategies such as supervised consumption sites to address overdose morbidity and mortality, as these structural solutions may appeal to a sample of participants from a syringe services program (36). Sitting in Rhode's proposed framework, these potential interventions address the environment–whether physical, social, economic or policy–and the level of environmental influence–micro or macro–in which people think about and potentially act on harm reduction (34).

Importantly, most knowledge of fentanyl, including its effects and dangers, appeared to be from participants' personal and peer experiences with use rather than reliance on public health information or resources despite participants having proximity to the vast educational and prevention infrastructure of the SSP. Participants shared what they believed to be personal adverse consequences of fentanyl use, but many demonstrated a lack of understanding of why/how those consequences occurred and expressed a desire to have more information available to know the effects of fentanyl on the body, especially before they started use. Although public health information resources such as the Centers for Disease Control and Prevention website exist, (37) the ease of access and presentation of data did not have great relevance to participants, whereas the experiences and knowledge of peers with fentanyl and test strips were recalled by the sample, indicating that using peer educators this may be an important way to deliver information (38). The salience of health education materials is further reduced when delivered in the context of the location, in this case Philadelphia, where fentanyl is nearly unavoidable (6). Findings serve as a cogent example of the importance of public health professionals understanding the trends of regional drug markets because this has implications for the content and delivery of harm reduction messaging strategies.

Our results also indicate diminished agency to act on health-protective behaviors, like using fentanyl test strips. These attitudes are consistent with current literature which identifies a higher perceived risk of fentanyl overdose among individuals who use fentanyl or other opioids but aligns with a sense of hopelessness about personal use and a lack of interest in fentanyl test strips to decrease risk of lethal overdose or significant harm/injury (21–23). However, research has also found acceptance and uptake of test strips by this population (24, 39, 40), including in Philadelphia (24). Our discrepant findings, particularly with Reed et al.'s work also in Philadelphia (24), may be attributable to different sampling strategies; Reed et al. recruited participants who reported past use of test strips for substances other than heroin whereas this was not required of this sample. Thus, it makes sense that Reed et al.'s sample reported interest in test strips whereas those participating in this study were more variable in attitudes toward test strips. This study sample was also socioeconomically lower-resourced and discussed economic hardships that were exacerbated by COVID-19, which may have affected attitudes. Similarly, Goldman et al. (39) and Park et al. (41) reported findings from participants that had agreed to participate in a fentanyl test strip intervention, which may have biased results toward favorability of test strips. Results presented here did not suggest that test strips are not viable; clearly these other investigations provide clear and actionable foundations for scale-up of fentanyl test strip focused interventions. Instead, these findings are a reminder of a person-first approach to harm reduction uptake interventions that must meet people where they are. Moreover, the fentanyl-saturated Philadelphian drug market (42), coupled with reduced economic means to purchase fentanyl-free drugs, may also contribute to the devaluation of fentanyl test strips observed in this sample if you are not already using them. In regions like Philadelphia, fentanyl test strips may best be presented to people who use drugs as a signaling tool to have other resources available such as having multiple doses of naloxone available before using substances known to contain fentanyl.

The COVID-19 pandemic has also certainly exacerbated the proliferation of fentanyl-adulterated products and created new mental health challenges. The social and psychological risks of the pandemic have been shown to potentially intensify drug use and increase feelings of hopelessness (43). Our respondents discussed how hard isolation and potential loss of income can be for those using drugs. This, coupled with escalating street prices due to a limited drug supply brought on by importation and transportation issues during the epidemic, (44) made locally produced fentanyl more available in a variety of drugs, increasing overdose risk (45). Emerging research on the impact of the pandemic on people who use drugs has shown that the effects of quarantine, isolation, mandates on social distancing, and temporary closure of public facilities and organizations, may have also exacerbated overdose risk (46). Social distancing may worsen overdose risk by reducing proximity to peers who potentially could reverse an overdose with naloxone, (47) which may have contributed to the rise in overdose responses by emergency medical services during the pandemic. (8) Closures and other mitigation strategies may have also undermined access to treatment and recovery services, further magnifying health disparities (48). Another effect of social distancing was the effect on access to harm reduction services, futher exacerbating feelings of isolation (47). Bolinski et al. (49) showed that in a qualitative study with people who use drugs in rural Illinois that isolation from others caused not only increased drug use but feelings of depression, anxiety and loneliness. Though different geographically than those in this study, similar comments by participants on drug use and the effect of COVID-19 mitigation on mental health were observed. This and the findings from other emerging research could inform strategies to prevent a surge in overdose occurrence and deaths, as well mental health effects, during public health emergencies similar to that posed by the COVID-19 pandemic.

Another escalating problem exacerbated by the COVID-19 pandemic is the presence of fentanyl not only in opioids such as heroin, but also stimulants such as cocaine and methamphetamine (12, 13, 50). This is certainly the case in Philadelphia (6, 7) and was articulated by our participants, underscoring the need for intervention. The increasing prevalence of fentanyl in drug supplies may undercut the low perceived importance of fentanyl test strips observed in our sample; if fentanyl is nearly unavoidable, why test for it? But the increasing adulteration of other substances with fentanyl makes it vitally important to use harm reduction strategies to help people who use drugs avoid overdose or death due to fentanyl. Participants felt having information about fentanyl's effects on the body, provided in a culturally salient way, was key to addressing the rising overdose rate. Thus, future work should explore how interventions may optimize information and enhance self-efficacy toward acting on harm reduction strategies within the context of the risk environment. Previous interventions to bolster self-efficacy among those who use drugs, however, have been mixed (51). Attention to the timing of delivery of information on prevention and strategies on how best to deliver that information to people using fentanyl to promote the integration of that information into existing harm reduction strategies and trusted environment would be important components to increasing agency and self-efficacy.

Finally, it should be noted that there are significant limitations to the availability of effective treatment for fentanyl's toxic effects (52). While naloxone is the only drug approved by the FDA to treat opioid overdose and specifically respiratory depression, (53, 54) there are currently no FDA approved drugs available to non-medical personnel in a community setting to treat vocal cord closure and chest wall rigidity caused by fentanyl or fentanyl analog overdoses (55). In the absence of these treatments, community agencies, such as SSPs, should think about how to better integrate other preventive measures. But many of these harm reduction methods are not feasible or accepted in drug using communities, as evidenced by studies on the carrying and use of naloxone. A cross-sectional study of people who use drugs from Baltimore found that knowledge of and training on how to use naloxone was high, but carrying it was low due to fear of the police or having to go to a healthcare setting that does not manage withdrawal (56). This has been seen in other locations as well (57, 58). In Philadelphia, during the time these interviews were conducted, possessing fentanyl test strips could be reason for arrest. An executive order by the mayor in August 2021 decriminalized the possession of test strips, acknowledging that use of them could help curb overdose (59). So, while harm reduction strategies exist, it can be difficult to get beyond both tangible and perceptual barriers. As a result, there appears to be a greater urgency in identifying and developing effective interventions for these vulnerable populations to increase understanding and perceived risk of the dangers of fentanyl to bridge that chasm.

### Limitations and future steps

Although these interviews provided us with new information about the perceptions and experiences of fentanyl use in a population that used drugs, the interviews were intentionally narrow in scope, and may not have reflected all harm reduction strategies the sample practiced. We asked these questions to this population of SSP participants because they had better access to fentanyl and services for overdose prevention than many other groups, making their perspectives informative for future intervention efforts. A limitation may have been the sample size. However, a systematic review suggests that data adequacy and saturation are the primary factors to be considered and that a sample size of 20 is adequate; (60) This sample meets both standards. Finally, to develop a robust understanding of attitudes toward risks and effects of fentanyl use across the country, it will be imperative to gather similar data in additional, varied (urban and rural) settings. This basic level of knowledge will provide a more solid foundation for an effective intervention with broader appeal and target audience.

### Conclusions

In addition to gathering preliminary data to assess the perceptions and experiences of fentanyl use risks during the COVID-19 pandemic, utilizing a participatory approach to develop, implement, and evaluate an opioid education intervention that incorporates information about fentanyl could be an effective and novel step to increase awareness about its risks and adverse consequences of use. For now, creating greater awareness of fentanyl toxicity effects and increasing self-efficacy in being able to protect from overdose and death could be a proactive step in saving lives.

## Data availability statement

The raw data supporting the conclusions of this article will be made available by the authors, without undue reservation.

## Ethics statement

The studies involving human participants were reviewed and approved by Temple University Institutional Review Board. Written informed consent for participation was not required for this study in accordance with the national legislation and the institutional requirements.

## Author contributions

SB: conceptualization, methodology, resources, writing–original draft, writing–review and editing, visualization, supervision, and funding acquisition. PK: conceptualization, formal analysis, data curation, writing–review and editing, and project administration. SP-K: data curation and writing–original draft. JP: data curation. KM: formal analysis and writing–original draft. EL: conceptualization. JW: conceptualization, writing–review and editing, and supervision. PT: conceptualization and writing–review and editing. All authors read and approved the final manuscript.

## Funding

This work was funded by the National Institute on Drug Abuse (3 R34 DA046305-03S1; SB PI). The funding body played no role in the design of the study and collection, analysis, and interpretation of data and in writing the manuscript.

## Conflict of interest

SP-K, KM, EL, JW, and PT were employed by CODA, Inc. The remaining authors declare that the research was conducted in the absence of any commercial or financial relationships that could be construed as a potential conflict of interest.

## Publisher's Note

All claims expressed in this article are solely those of the authors and do not necessarily represent those of their affiliated organizations, or those of the publisher, the editors and the reviewers. Any product that may be evaluated in this article, or claim that may be made by its manufacturer, is not guaranteed or endorsed by the publisher.

## References

[1] Centers for Disease Control Prevention National Center for Injury Prevention Control. Drug Overdose Deaths. (2021). Available online at: https://www.cdc.gov/drugoverdose/deaths/index.html (accessed November 03, 2021).

[2] Hedegaard H Bastian BA Trinidad JP Spencer M Warner M. Drugs most frequently involved in drug overdose deaths: United States, 2011 – 2016. National Vital Statistics Reports, Vol. 67, Hyattsville, MD: National Center for Health Statistics (2018). 30707673

[3] Wilson N Kariisa M Seth P Smith H IV Davis NL. Drug and opioid-involved overdose deaths – United States, 2017-2018. MMWR Morb Mortal Wkly Rep. (2020) 69:11. 10.15585/mmwr.mm6911a4 32191688 PMC7739981

[4] O'Donnell J Gladden RM Mattson CL Hunter CT Davis NL. Vital signs: characteristics of drug overdose deaths involving opioids and stimulants – 24 states and the District of Columbia, January – June 2019. MMWR Morb Mortal Wkly Rep. (2020) 69:35. 10.15585/mmwr.mm6935a1 32881854 PMC7470457

[5] Centers for Disease Control Prevention,. Drug Overdose Deaths in the U.S. Top 100,000 Annually. (2021). Available online at: https://www.cdc.gov/nchs/pressroom/nchs_press_releases/2021/20211117.htm (accessed January 23, 2022).

[6] Philadelphia Department of Public Health. Unintentional drug overdose fatalities in Philadelphia, 2020. (2021). Available online at: https://www.phila.gov/media/20210603100151/CHARTv6e5.pdf. [Accessed November 03, 2021].

[7] Philadelphia Department of Public Health. Substance Use Philadelphia, Unintentional Overdose Deaths. (2021). Available online at: https://www.substanceusephilly.com/unintentional-overdose-deaths (accessed June 22, 2022).

[8] Khoury D Preiss A Geiger P Anwar M Conway KP. Increases in Naloxone administrations by emergency medical services providers during the COVID-19 pandemic: retrospective time series study. JMIR Public Health Surveill. (2021) 7:5. E29298 10.2196/29298 33999828 PMC8163496

[9] Volkow ND. Collision of the COVID-19 and addiction epidemics. Ann Intern Med. (2020) 173:1 p. 61-2. 10.7326/M20-1212 PMC7138334 32240293

[10] Shover CL Falasinnu TO Dwyer CL Santos NB Cunningham NJ Freedman. Steep increased in fentanyl-related mortality west of the Mississippi River: synthesizing recent evidence from county and state surveillance. Drug Alcohol Depend. (2020) 216:108314. 10.1016/j.drugalcdep.2020.108314 33038637 PMC7521591

[11] Vestal C. As the opioid crisis peaks, meth and cocaine deaths explode. PEW. (2019) Available online at: https://www.pewtrusts.org/en/research-and-analysis/blogs/stateline/2019/05/13/as-the-opioid-crisis-peaks-meth-and-cocaine-deaths-explode (accessed November 03, 2021)].

[12] Twillman RK Dawson E LaRue L Guevara MG Whitley P Huskey A. Evaluation of trends of near-real-time urine drug test results for methamphetamine, cocaine, heroin, and fentanyl. JAMA Netw Open. (2020) 3:e1918514; 10.1001/jamanetworkopen.2019.18514 PMC6991312 31899527

[13] Larue L Twillman RK Dawson E. Rate of fentanyl positivity among urine drug test results positive for cocaine or methamphetamine. JAMA Netw Open. (2019) 2:e192851; 10.1001/jamanetworkopen.2019.2851 PMC6487565 31026029

[14] Bennet J Abrams J Riper DV Horrow J. Difficult or impossible ventilation after Sufentanil-induced anesthesia is caused primarily by vocal cord closure. Anesthesiology. (1997) 87:5. 10.1097/00000542-199711000-00010 9366458

[15] Scamman FL. Fentanyl O2-N2O rigidity and pulmonary compliance. Anesth Analg. (1983) 62:332–4. 10.1213/00000539-198303000-00008 6829933

[16] Streisand J Bailey P Lemaire L Ashburn MA Tarver SD Varvel J. Fentanyl-induced rigidity and unconsciousness in human volunteers. Incidence, duration, and plasma concentrations. Anesthesiology. (1993) 78:4. 10.1097/00000542-199304000-00003 8466061

[17] Marco CA Trautman W Cook A Mann D Rasp J Perkins O. Naloxone use among emergency department patients with opioid overdose. J Emerg Med. (2018) 55:64–70. 10.1016/j.jemermed.2018.04.022 29776702

[18] Klebacher R Harris MI Ariyaprakai N Tagore A Robbins V Dudley LS. Incidence of Naloxone redosing in the age of the new opioid epidemic. Prehosp Emerg Care. (2017) 21:682–7. 10.1080/10903127.2017.1335818 28686547

[19] Moss RB Carlo DJ. Higher doses of naloxone are needed in the synthetic opioid era. Substance Abuse Treatment, Prevention, and Policy. (2019) 14:6. 10.1186/s13011-019-0195-4 30777088 PMC6379922

[20] National Institute on Drug Abuse. Fentanyl Drug Facts. (2021). Available online at: https://www.drugabuse.gov/publications/drugfacts/fentanyl (accessed February 21, 2022).

[21] Stein MD Kenney SR Anderson BJ Bailey GL. Perceptions about fentanyl-adulterated heroin and overdose risk reduction behaviors among persons seeking treatment for heroin use. J Subst Abuse Treat. (2019) 104:144–7. 10.1016/j.jsat.2019.07.006 31370978 PMC6702957

[22] Latkin CA Dayton L Davey-Rothwell MA Tobin KE. Fentanyl and drug overdose: perceptions of fentanyl risk, overdose risk behaviors, and opportunities for intervention among people who use opioids in Baltimore, USA. Subst Use Misuse. (2019) 54:6. 10.1080/10826084.2018.1555597 30767590 PMC6476669

[23] Ciccarone D Ondocsin J Mars SG. Heroin uncertainties: exploring users' perceptions of fentanyl-adulterated and-substituted ‘heroin'. Int J Drug Pol. (2017) 45:146–55. 10.1016/j.drugpo.2017.06.004 28735775 PMC5577861

[24] Reed MK Salcedo VJ Guth A Rising KL. “If I had them I would use them every time”: perspectives on fentanyl test strip use from people who use drugs. J Subst Abuse Treat. (2022) 108790. 10.1016/j.jsat.2022.108790 35577663

[25] Rouhani S Park JN Morales KB Green TC Sherman SG. Harm reduction measures employed by people using opioids with suspected fentanyl exposure in Boston, Baltimore, and Providence. Harm Reduct J. (2019) 16:39. 10.1186/s12954-019-0311-9 31234942 PMC6591810

[26] Szalavitz M. Undoing Drugs: The Untold Story of Harm Reduction and The Future of Addiction (1st ed.). Hachette Book Group. (2021).

[27] Torralva R Janowsky A. Noradrenergic mechanisms in fentanyl-mediated rapid death explain failure of Naloxone in the opioid crisis. J Pharmacol Exp Ther. (2019) 371:453–75. 10.1124/jpet.119.258566 31492824 PMC6863461

[28] Ball AL. HIV, injecting drug use and harm reduction: a public health response. Addiction. (2007) 102:684–90. 10.1111/j.1360-0443.2007.01761.x 17506148

[29] Jessop A Bass SB Gashat M Guttierez M. Perceptions of barriers and benefits of HCV treatment and correlates to treatment intention in methadone patients. J Healthcare Poor Underserved. (2019) 30:4. 10.1353/hpu.2019.0094 31680107

[30] Cook RR Torralva R King C Lum PJ Tookes H Foot C. Associations between fentanyl use and initiation, persistence, and retention on medications for opioid use disorder among people living with uncontrolled HIV disease. Drug Alcohol Depend. (2021) 228:109077. 10.1016/j.drugalcdep.2021.109077 34600253 PMC8595584

[31] Wisdom JP Manuel JI Drake RE. Substance use disorder among people with first-episode psychosis: A systematic review of course and treatment. Psychiatric Services. (2011) 62:1007–1012. 10.1176/ps.62.9.pss6209_1007 21885577 PMC3575521

[32] Guest G McQueen KM Namey EE. Applied Thematic Analysis. SAGE Publications, Inc. (2012). 10.4135/9781483384436

[33] Braun V Clarke V. Using thematic analysis in psychology. Qual Res Psychol. (2006) 3:77–101. 10.1191/1478088706qp063oa

[34] Rhodes T. Risk environments and drug harms: A social science for harm reduction approach. Int J Drug Policy. (2009) 20:193–201. 10.1016/j.drugpo.2008.10.003 19147339

[35] Bonar EE Rosenberg H. Using the health belief model to predict injecting drug users' intentions to employ harm reduction strategies. Addict Behav. (2011) 36:11. 10.1016/j.addbeh.2011.06.010 21763076

[36] Rubin R Suran M. Supervised consumption sites—a tool for reducing risk of overdose deaths and infectious diseases in people who use illicit drugs. JAMA. (2022) 327:1532–34. 10.1001/jama.2022.4017 35385054

[37] Centers for Disease Control Prevention. Fentanyl. (2021) Available online at: https://www.cdc.gov/opioids/basics/fentanyl.html (accessed February 21, 2022).

[38] Mercer F Miler JA Pauly B Carver H Hnízdilová K Foster R. Peer support and overdose prevention responses: a systematic ‘state-of-the-art' review. Int J Environ Res Public Health. (2021) 18:12073. 10.3390/ijerph182212073 34831839 PMC8621858

[39] Goldman JE Waye KM Pereira KA Krieger MS Yedinak JL Marshal BDL. Perspectives on rapid fentanyl test strips as a harm reduction practice among young adults who use drugs: a qualitative study. Harm Reduct J. (2019) 16:3. 10.1186/s12954-018-0276-0 30621699 PMC6325714

[40] Reed MK Guth A Salcedo VJ Hom JK Rising KL. “You can't go wrong being safe”: Motivations, patterns, and context surrounding use of fentanyl test strips for heroin and other drugs. Int J Drug Pol. (2022) 103:103643. 10.1016/j.drugpo.2022.103643 35255392

[41] Park JN Frankel S Morris M Dieni O Fahey-Morrison L Luta M. Evaluation of fentanyl test strip distribution in two mid-Atlantic syringe services programs. Int J Drug Policy. (2021) 94:103196. 10.1016/j.drugpo.2021.103196 33713964

[42] Ornell F Moura HF Scherer JN Pechansky F Kessler FHP von Diemen L. The COVID-19 pandemic and its impact on substance use: implications for prevention and treatment. Psychiatry Res. (2020) 289:113096. 10.1016/j.psychres.2020.113096 32405115 PMC7219362

[43] United Nations,. COVID-19 Causes Some Illegal Drug Prices to Surge, as Supplies are Disrupted Worldwide. (2020). Available online at: https://news.un.org/en/story/2020/05/1063512 (accessed November 03, 2021).

[44] Whelan A. As COVID-19 spikes, so do overdoses among Center City restaurant workers, jolting a stressed community. Philadelphia Inquirer. (2020) Available online at: https://www.inquirer.com/health/opioid-addiction/overdose-spike-restaurant-industry-center-city-20201128.html (accessed November 03, 2021).

[45] Lin C Clingan SE Cousins SJ Valdez J Mooney LJ Hser Y. The impact of COVID-19 on substance use disorder treatment in California: Service providers' perspectives. J Subst Abuse Treat. (2022) 133:108544. 10.1016/j.jsat.2021.108544 34183213 PMC8702565

[46] Linas BP Savinkina A Barbosa Mueller PP Cerda M keyes K. A clash of epidemics: Impact of the COVID-19 pandemic response on opioid overdose. J Subst Abuse Treat. (2021) 120:108158. 10.1016/j.jsat.2020.108158 33298298 PMC7536128

[47] Nguyen T Buxton JA. Pathways between COVID-19 public health responses and increasing overdose risks: A rapid review and conceptual framework. Int J Drug Policy. (2021) 93:103236. 10.1016/j.drugpo.2021.103236 33838990 PMC8545050

[48] Fleming T Barker A Ivsins A Vakharia S McNeil R. Stimulant safe supply: a potential opportunity to respond to the overdose epidemic. Harm Reduct J. (2020) 17:1. 10.1186/s12954-019-0351-1 31924209 PMC6954588

[49] Bolinski RS Walters S Salisbury-Afshar E Ouellet LJ Jenkins WD Almirol E. The impact of the COVID-19 pandemic on drug use behaviors, fentanyl exposure, and harm reduction service support among people who use drugs in rural settings. Intl J Environ Res Public Health. (2022) 19:2230. 10.3390/ijerph19042230 35206421 PMC8872091

[50] Centers for Disease Control and Prevention. Overdose deaths accelerating during COVID-19: expanded prevention efforts needed. (2020). Available online at: https://www.cdc.gov/media/releases/2020/p1218-overdose-deaths-covid-19.html (accessed November 03 2021).

[51] Kadden RM Litt MD. The role of self-efficacy in the treatment of substance use disorders. Addict Behav. (2011) 36:12. 10.1016/j.addbeh.2011.07.032 21849232 PMC3179802

[52] Comer SD Cahill CM. Fentanyl: receptor pharmacology, abuse potential, and implications for treatment. Neurosci Biobehav Rev. (2018) 106:49–57. 10.1016/j.neubiorev.2018.12.005 30528374 PMC7233332

[53] National Institute on Drug Abuse. Naloxone for Opioid Overdose: Life-Saving Science (2021). Available online at: https://www.drugabuse.gov/publications/naloxone-opioid-overdose-life-saving-science (accessed November 03, 2021).

[54] U.S. Food and Drug Administration. FDA Approves Higher Dosage of Naloxone Nasal Spray to Treat Opioid Overdose. (2021). Available online at: https://www.fda.gov/news-events/press-announcements/fda-approves-higher-dosage-naloxone-nasal-spray-treat-opioid-overdose (accessed November 03 2021).]

[55] Baumann MH Kopajtic TA Madras BK. Pharmacological research as a key component in mitigating the opioid overdose crisis. Trends Pharmacol Sci. (2018) 39:12. 10.1016/j.tips.2018.09.006 30454770

[56] Walley AY Xuan Z Hackman HH Quinn E Doe-Simkins M Sorensen-Alawad A. Opioid overdose rates and implementation of overdose education and nasal naloxone distribution in Massachusetts: interrupted time series analysis. BMJ. (2013) 346:f174. 10.1136/bmj.f174 23372174 PMC4688551

[57] Seal KH Downing M Kral AH. Attitudes about prescribing take-home naloxone to injection drug users for the management of heroin overdose: a survey of street-recruited injectors in the San Francisco Bay Area. J Urban Health. (2003) 80:291–301. 10.1093/jurban/jtg032 12791805 PMC3456285

[58] Koester S Mueller SR Raville L Langegger S Binswanger IA. Why are some people who have received overdose education and naloxone reticent to call Emergency Medical Services in the event of overdose? Int J Drug Policy. (2017) 48:115–24. 10.1016/j.drugpo.2017.06.008 28734745 PMC5825210

[59] City City of Philadelphia Office Office of the Mayor. Executive Order 4-21 (2020). Available online at: https://www.phila.gov/media/20210802122151/Executive-Order-4-21.pdf (accessed November 03, 2021).

[60] Vasileiou K Barnett J Thorpe S Young T. Characterising and justifying sample size sufficiency in interview-based studies: systematic analysis of qualitative health research over a 15-year period. BMC Med Res Methodol. (2018) 18:148. 10.1186/s12874-018-0594-7 30463515 PMC6249736

